# Adult-onset combined methylmalonic acidemia and hyperhomocysteinemia, cblC type with aortic dissection and acute kidney injury: a case report

**DOI:** 10.1186/s12882-023-03414-9

**Published:** 2024-01-04

**Authors:** Qiufa Hao, Bei Jiang, Yuying Zhao, Zhao Hu

**Affiliations:** 1 Department of Nephrology, Qilu Hospital, Cheeloo College of Medicine, Shandong University, No. 107 West Wenhua Road, Jinan, Shandong Province 250012 China; 2 Research Institute of Neuromuscular and Neurodegenerative Diseases and Department of Neurology, Qilu Hospital, Cheeloo College of Medicine, Shandong University, No. 107 West Wenhua Road, Jinan, Shandong Province 250012 China

**Keywords:** Methylmalonic acidemia, Hyperhomocysteinemia, Cobalamin C deficiency, Aortic Dissection, Acute kidney injury

## Abstract

**Background:**

Combined methylmalonic acidemia (MMA) and hyperhomocysteinemia, cobalamin C (cblC) type, also named cblC deficiency is a rare autosomal recessive genetic metabolic disease. It progressively causes neurological, hematologic, renal and other system dysfunction. The clinical manifestations are relatively different due to the onset time of disease.

**Case presentation:**

This report describes a rare case of a 26 year old man with cblC deficiency who developed life-threatening aortic dissection and acute kidney injury (AKI) and showed neuropsychiatric symptoms with elevated serum homocysteine and methylmalonic aciduria. After emergent operation and intramuscular cobalamin supplementation therapy, the male recovered from aortic dissection, neurological disorder and AKI. Finally, two previously published compound heterozygous variants, c.482G > A (p.R161Q) and c.658_660del (p.K220del) in the *MMACHC* gene were detected in this patient and he was confirmed to have cblC deficiency.

**Conclusions:**

Poor cognizance of presenting symptoms and biochemical features of adult onset cblC disease may cause delayed diagnosis and management. This case is the first to depict a case of adult-onset cblC deficiency with aortic dissection. This clinical finding may contribute to the diagnosis of cblC deficiency.

## Background

Combined methylmalonic acidemia (MMA) and homocystinemia, cobalamin C (cblC) type, also named cblC deficiency is a metabolic disease with errors in vitamin B12 (cobalamin) synthesis [1, 2]. The disease is caused by variants of the *MMACHC* gene and is transmitted as an autosomal recessive trait [3]. The defect decreases the conversion of vitamin B12 to methylcobalamin and adenosylcobalamin, which will lead to high levels of serum MMA and homocysteine. Adult-onset cblC deficiency usually manifests itself as neuropsychiatric symptoms, progressive cognitive decline, hematological manifestations and renal dysfunction [3]. The manifestations of cblC deficiency are highly variable and the diagnosis is challenging. However, the prognosis is relatively good with proper treatment. Early diagnosis and treatment are important. Here we describe a case of adult-onset cblC deficiency with aortic dissection and AKI. This is the first study to depict a case of cblC deficiency with aortic dissection as a presenting sign.

## Case presentation

**Fig. 1:**
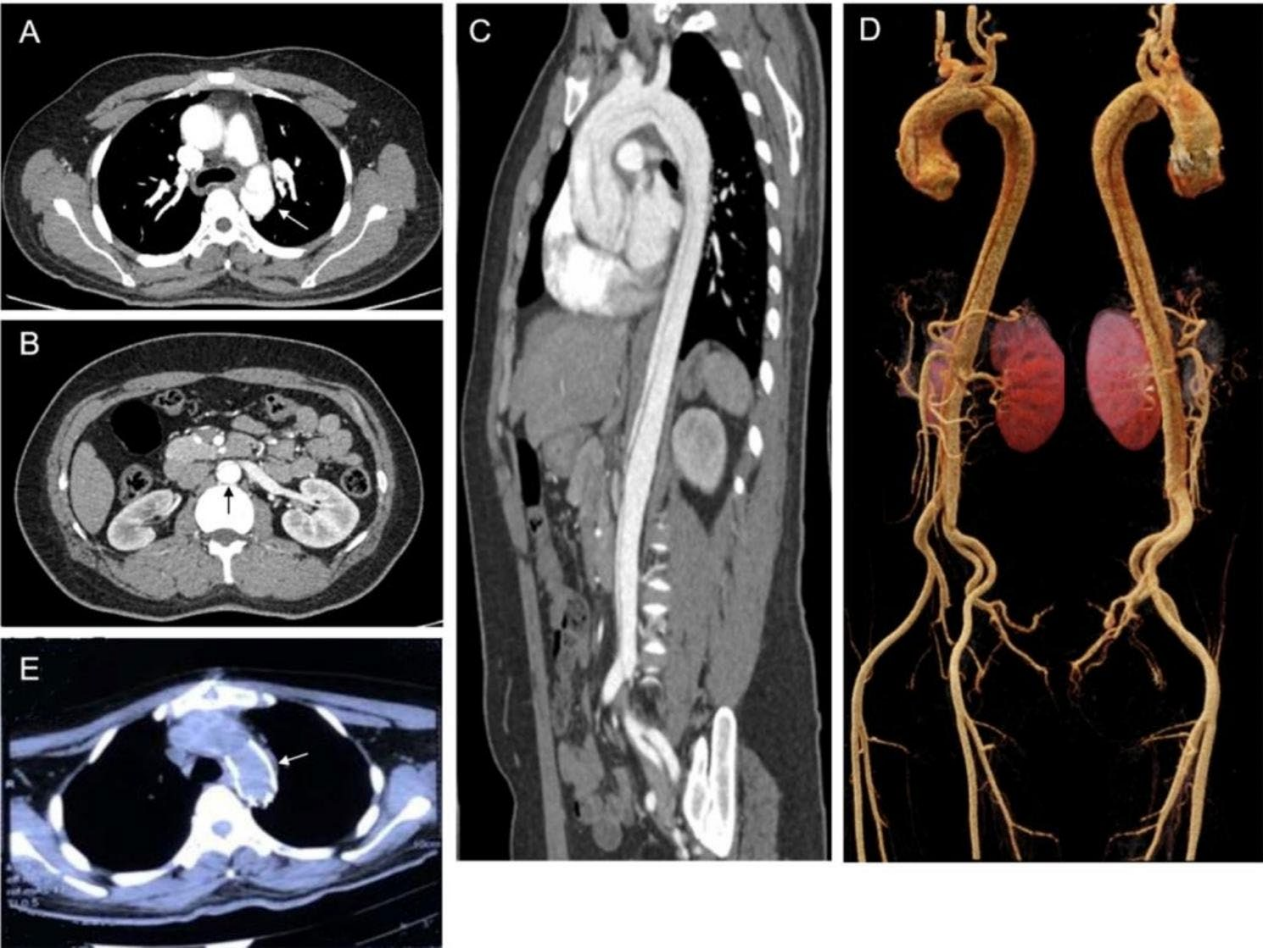
Images of aortic dissection in the patient before and after the operation. (**A**) Contrast-enhanced CT image of aortic dissection in the aortic arch. (**B**) Contrast-enhanced CT image of aortic dissection in the abdominal aorta. (**C**) CT angiography of aortic dissection on sagittal reconstruction. (**D**) Three-dimensional reconstruction of the aorta demonstrating aortic dissection (Stanford type A). (**E**) CT image of the aortic arch after the operation. (The arrow represents the position of the lesion in the aorta.)

A 26-year-old man was admitted to Shandong Provincial Hospital for sudden squeezing chest and abdominal pain for 1 day. On physical examination, his blood pressure was 147/87 mmHg and heart rate was regular at 95 beats pre minute. He was diagnosed with aortic dissection (Stanford type A) and then underwent an operation (Fig. 1). Meanwhile, he suffered AKI due to renal artery involvement in aortic dissection and his serum creatinine increased from 180.1 µmol/L to 579.7 µmol/L (62–115 µmol/L) a week later. The operation was successful and his serum creatinine level was maintained at 200–300 µmol/L. Then he was discharged from the hospital. Three months after the operation, he gradually demonstrated anorexia and lost interest in the outside things. Increasingly, he was emotionally unstable and behaved abnormally. He sometimes abused his parents or kept speaking. Then, he went to a doctor in Shandong Provincial Mental Health Center and was diagnosed with a substupor state. His symptoms were partially improved with treatment of olanzapine, sulpiride, and sertraline. Approximately 1.5 months later, his symptoms worsened. He didn’t communicate with others and he had some hallucinations. Then, he was transferred to the neurology department of Qilu hospital. His expression was dim, and his bilateral legs had positiveBabinski and Chadcock signs. Liver and kidney function tests showed serum homocysteine 481.7 µmol/L (< 15 µmol/L) and serum creatinine 326 µmol/L. Antinuclear antibody spectrum, thyroid function, serum ceruloplasmin, blood ammonia and TORCH screen results were normal. Cerebral magnetic resonance imaging and magnetic resonance angiography showed multiple intracranial ischemia areas; multiple cerebral arteriosclerosis and stenosis. To determine the reason for hyperhomocysteinemia, we further examined urinary organic acids by gas-chromatography mass spectrometry and metabolite detection related to carnitine in blood by tandem mass spectrometry. Urinary organic acid analysis showed methylmalonic acid 171.55 mmol/molCr (0.3–3.6 mmol/molCr), 3-hydroxypropionic acid 5.5 mmol/molCr (0.2–1.1 mmol/molCr) and malonic acid 1.31 mmol/molCr (0-0.1 mmol/molCr). The ratio of propionylcarnitine (C3)/free carnitine (C0) in blood was 0.57 (0.02–0.25). The level of vitamin B12 was 1232 pg/ml (243–894 pg/ml). We examined targeted next generation sequencing (NGS) panel in the patient.

**Fig. 2:**
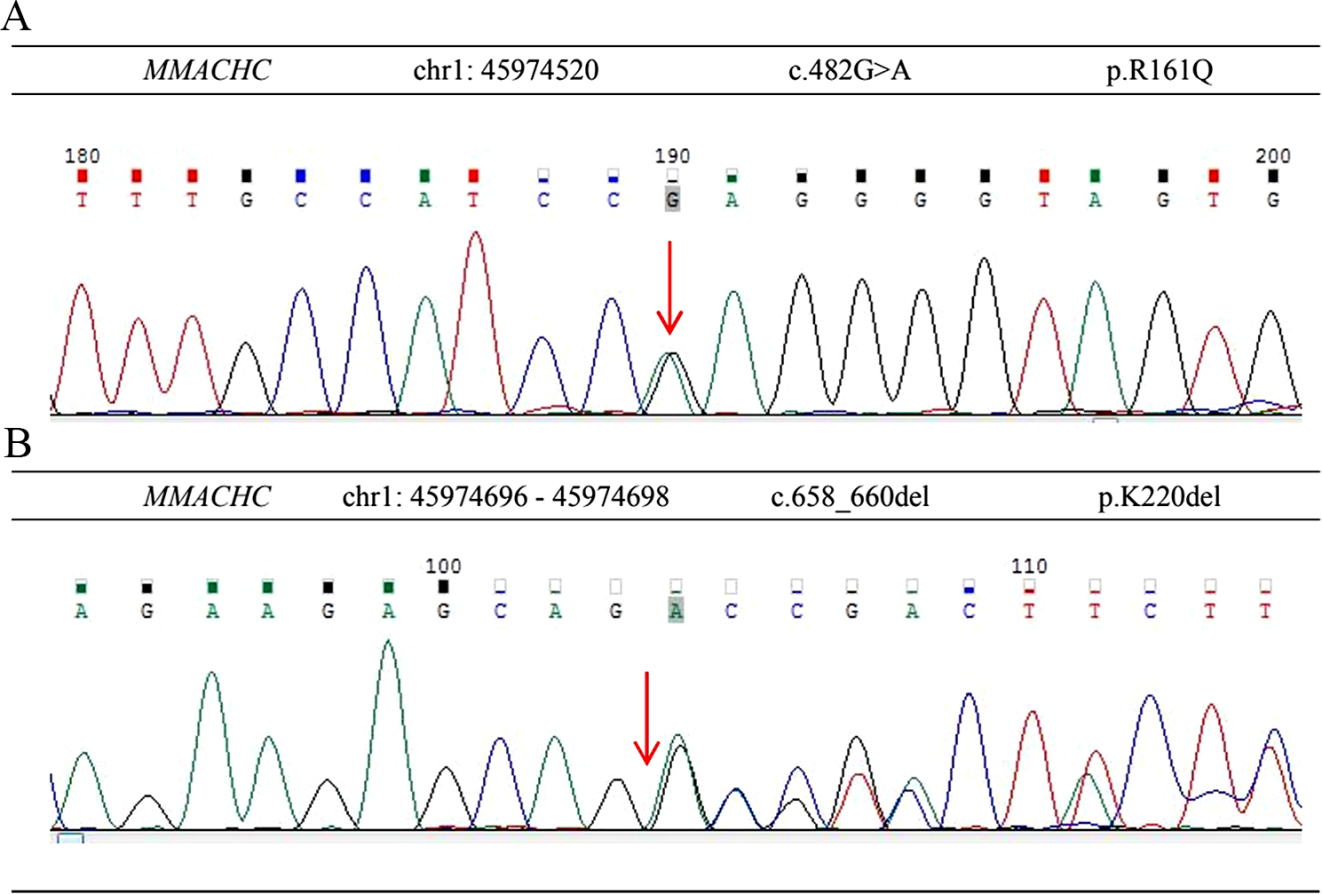
The *MMACHC* gene mutation sites of the patient. (**A**) The *MMACHC* gene c.482G > A (p. R161Q). (**B**) The *MMACHC* gene c.658_660del (p. K220del). (The arrow represents the gene mutation sites.)

.Two compound heterogeneous pathogenic mutations were further identified in the *MMACHC* gene namely c.482G > A (p. R161Q) and c.658_660del (p. K220del) (Fig. 2). The results of NGS panel indicated no suspicious pathogenic genes inducing aortic dissection. These results suggested that the diagnosis was combined MMA and homocystinemia, cblC type. He was treated with hydroxycobalamin, betaine, folinate, L-carnitine and symptomatic treatment for kidney dysfunction. After 4 months, the male had an outpatient visit and his neuropsychiatric symptoms significantly improved. Liver and kidney function tests showed homocysteine 48.7 µmol/L and serum creatinine 141 µmol/L. The urine routine examination showed urinary protein and occult blood is negative.

## Discussion and conclusions

The cblC deficiency is a common type of cobalamin metabolic disorder. The cblC deficiency is the most common type of MMA in China [4]. The cblC deficiency patients could present anorexia, progressive encephalopathy, renal dysfunction and hematologic abnormalities [3]. Early diagnosis is very important, which is underscored by gradual progression of the impaired system. The cblC deficiency is triggered by mutation in the *MMACHC* gene. More than 100 mutations in the *MMACHC* gene have been reported up to now [5].In this patient, we found 2 heterozygous mutations: c.482G > A (p. R161Q) and c.658_660del (p. K220del). These 2 mutations have been reported in previous cases [6, 7]. The *MMACHC* gene mutation c.482G > A has been repeatedly reported with late-onset presentation [3]. Adult-onset cblC deficiency was found the first onset symptoms at the age after 18 years old. In adult-onset cblC deficiency patients, neurological symptoms, isolated psychiatric symptoms and renal involvement are main onset symptoms. With the progress of disease, the manifestations related to neuropathy and cognitive decline are common symptoms. Vascular disease (thromboembolic disease) and renal disease (proteinuria and renal failure) are also found in these patients [8]. The average level of serum homocysteine at diagnosis was 137.4 µmol/L (27.9–288 µmol/L) [8]. The level of serum homocysteine in this patient (481.7 µmol/L) was much higher than the average level. The treatment results in adult-onset patients are always with marked improvement. We need to raise awareness for this rare but treatable disease.

The cblC deficiency has been related to neurocognitive and vascular disorders. Thromboembolic complications including recurrent venous thrombosis, pulmonary thrombosis, cor pulmonale and cerebrovascular complications are important conditions in cblC deficiency patients [1]. Thrombotic microangiopathy and pulmonary arterial hypertension presented in cblC deficiency patients [9, 10]. Wide vascular lesions, such as arteriosclerosis, also presented in these patients [11]. Endothelial dysfunction has been regarded as an important pathogenesis of thrombotic microangiopathy, pulmonary arterial hypertension and arteriosclerosis. To the best of our knowledge, aortic dissection in cblC deficiency patients has not been reported. We may wonder what caused aortic dissection in a young man. The common risk factors of aortic dissection include hypertension, atherosclerosis, congenital diseases, trauma, inflammation, infection and others. The specific risk factors in this patient were male sex and stage 1 hypertension. He was healthy with no drugs previously. The targeted NGS panel indicated no suspicious heritable thoracic aortic diseases inducing aortic dissection. We suspected whether cblC deficiency caused aortic dissection. Hyperhomocysteinemia is a critical biomarker of the cblC deficiency. Epidemiological studies have suggested an association of hyperhomocysteinemia and aortic dissection, but discrepancies exist. It has been proven that hyperhomocysteinemia is a risk factor for arterial endothelial dysfunction [12]. 48% patients with abdominal aortic aneurysm were found hyperhomocysteinemia and the levels of plasma homocysteine were higher in patients than in control subjects [13]. In Marfan patients, severe cardiovascular manifestations and aortic dissection were found to be related with homocysteine plasma levels [14]. In addition, the levels of median plasma homocysteine were higher in patients with spontaneous cervical artery dissection than in control subjects [13]. Homocysteine plays a significant role in development of aortic dissection and homocysteinemia is a risk factor for aortic dissection. Impaired fibrillin deposition into extracellular matrix was found in aortic aneurysm and dissection. In the *FBN1* (the gene for fibrillin-1) mutation patients, reduced fibrillin-1 deposition into pericellular matrix formed weakness of elastic tissue, which could cause aortic aneurysms or dissections. Fibrillin-1 regarded as the important component in extracelluar connective tissue was susceptible to homocysteine attack and irreversible homocysteinylation of long-lived proteins should cause cumulative damage and progressive clinical manifestations [13]. Moreover, homocysteine could cause premature breakdown in arterial elastic fibers by activating the elastolytic activities [13]. Thus, we hypothesized that elevated homocysteinemia in this patient was closely associated with aortic dissection.

Renal disease and chronic kidney disease are considerable manifestations of cblC deficiency. Renal complications induced by cblC disease include tubulointerstitial nephritis, thrombotic microangiopathy, hemolytic uremic syndrome and proximal renal tubular acidosis [15, 16]. A study reviewed the kidney involvement in adult-onset cblC deficiency patients [8]. Glomerular disease, renal failure and hemolytic uremic syndrome were commonly found. Kidney biopsies in adult-onset patients usually showed the typical lesions of thrombotic microangiopathy, which was similar with renal damage in infancies with cblC deficies. However, the kidney dysfunction in this case was a process of AKI, caused by aortic dissection. Through symptomatic treatment, his kidney function greatly improved.

In conclusion, we demonstrated a case of cblC deficiency with aortic dissection. This may contribute to the diagnosis of cblC deficiency. It is important to pay more attention to the early diagnosis and treatment of cblC deficiency.

## Data Availability

The datasets analyzed during the current study are available from the corresponding author on reasonable request.

## Abbreviations

MMA: Methylmalonic academia
cblC: Cobalamin C
AKI: Acute kidney injury
